# Prognostic Value of Soluble Suppression of Tumorigenicity 2 in Chronic Kidney Disease Patients: A Meta-Analysis

**DOI:** 10.1155/2021/8881393

**Published:** 2021-01-25

**Authors:** Guangying Guo, Aoran Huang, Xin Huang, Tianhua Xu, Li Yao

**Affiliations:** Department of Nephrology, The First Hospital of China Medical University, 155 North Nanjing Street, Heping District, Shenyang 110000, China

## Abstract

**Objective:**

Previous studies have controversial results about the prognostic role of soluble suppression of tumorigenicity 2 (sST2) in chronic kidney disease (CKD). Therefore, we conduct this meta-analysis to access the association between sST2 and all-cause mortality, cardiovascular disease (CVD) mortality, and CVD events in patients with CKD.

**Methods:**

The publication studies on the association of sST2 with all-cause mortality, CVD mortality, and CVD events from PubMed and Embase were searched through August 2020. We pooled the hazard ratio (HR) comparing high versus low levels of sST2 and subgroup analysis based on treatment, continent, and diabetes mellitus (DM) proportion, and sample size was also performed.

**Results:**

There were 15 eligible studies with 11,063 CKD patients that were included in our meta-analysis. Elevated level of sST2 was associated with increased risk of all-cause mortality (HR 2.05; 95% confidence interval (CI), 1.51–2.78), CVD mortality (HR 1.68; 95% CI, 1.35–2.09), total CVD events (HR 1.88; 95% CI, 1.26–2.80), and HF (HR 1.35; 95% CI, 1.11–1.64). Subgroup analysis based on continent, DM percentage, and sample size showed that these factors did not influence the prognostic role of sST2 levels to all-cause mortality.

**Conclusions:**

Our results show that high levels of sST2 could predict the all-cause mortality, CVD mortality, and CVD events in CKD patients.

## 1. Introduction

Chronic kidney disease (CKD) results in great health burden with an approximate incidence rate of 9.1% worldwide. Especially in countries of low and middle income, the incidence rate of CKD is up to 14.1% [1, 2]. CKD not only has a huge effect on global health but also acts as an important risk factor for cardiovascular disease (CVD) which accounts for approximately 40% ~50% of deaths in adults when CKD progresses into end stage renal disease (ESRD) [1, 3]. Therefore, it is urgent to find biomarkers that could help identifying high-risk CKD patients and predicting the risk of mortality and CVD in CKD patients.

Suppression of tumorigenicity 2 (ST2) is a member of the interleukin-1 (IL-1) receptor family produced by various types of tissues and cells in response to inflammation, stress, and other triggers [4, 5]. There are two essential isoforms for ST2: a membrane linked form ST2L and a soluble form sST2 [6]. ST2L plays an important role in protecting heart muscle tissue from apoptosis, fibrosis, and cardiomyocyte hypertrophy through interaction with IL-33 which is a member of IL-1 cytokine family and a key ligand that binds to ST2L. In contrast, the sST2 decreases the cardioprotective effects of ST2L by competitively binding to IL-33 [4, 6]. Recent studies have evaluated sST2 as a prognosis marker in CKD patients independently of the renal function, age, and dialysis process, unlike other cardiac biomarkers [7, 8]. Wang et al. compared the role of sST2 with N-terminal probrain natriuretic peptide (NT-proBNP), a classical prognostic biomarker in CKD, on predicting both all-cause and cardiovascular mortality among maintenance hemodialysis (MHD) patients and found the superior role of sST2 [9]. However, another study found that sST2 alone did not predict prognosis and/or CVD events in nondialyzed CKD patients [8]. Therefore, a meta-analysis is required to systematically evaluate the prognostic value of sST2 in CKD patients.

The individual studies may have limitation to obtain a definitive conclusion. Considering the inconsistent results between the sST2 and adverse clinical outcomes, we conduct a meta-analysis by combining the results from all available studies to (1) evaluate the prognosis value of sST2 in CKD patients, (2) explore the potential between-study heterogeneity, and (3) investigate the potential publication bias.

## 2. Materials and Methods

### 2.1. Literature Search Strategy

Our meta-analysis was performed and reported according to the Preferred Reporting Items for Systematic Reviews and Meta-Analyses (PRISMA) statement [10]. We searched the literatures in PubMed and Excerpta Medica Database (Embase) from inception to 9 August 2020. The search strategy included a combination of the following keywords: (chronic kidney failure OR chronic renal failure OR chronic renal insufficiency OR chronic renal insufficiencies OR chronic kidney insufficiency OR chronic kidney insufficiencies OR chronic kidney disease OR chronic kidney diseases OR chronic renal disease OR chronic renal diseases OR end stage renal disease OR end-stage renal disease OR end stage kidney disease OR end-stage kidney disease OR end-stage renal failure OR dialysis OR renal dialyses OR hemodialysis OR hemodialyses OR extracorporeal dialyses OR kidney transplantation OR kidney transplantations OR renal transplantation OR renal transplantations OR kidney grafting) AND (ST2 OR sST2 OR suppression of tumorigenicity 2 OR interleukin 1 receptor like 1 protein). Moreover, we also reviewed the reference lists of all retrieved relevant articles and reviews to identify additional relevant studies that were not captured by our database searches.

### 2.2. Study Selection

Studies were included in present quantitative analysis if they meet the following criteria: (1) cohort studies (including prospective or retrospective designs); (2) the diagnostic criteria for CKD are based on definitions of the Kidney Disease Improving Global Outcomes (KDIGO) 2017 clinical practice guidelines [11]. (3) sST2 concentration at baseline was measured and provided the hazard ratio (HR) and the corresponding 95% confidence interval (CI) for sST2 concentration and adverse clinical outcomes and (4) reported at least one specific CVD events, such as CVD mortality, total CVD events, dysrhythmia, and heart failure (HF) or all-cause mortality using multivariable-adjusted risk evaluation. The following exclusion criteria were also used: (1) case reports, editorials, letters, or review articles; (2) patients were pregnant women or <18 years; and (3) HR or the corresponding 95% CI were not available. If data was duplicated in more than one study, we included the most recent one published. There were no restrictions on gender, race, CKD stage (including predialysis patients and those with ESRD who are receiving dialysis or functioning renal transplantations), or language of the studies.

To ensure the correct selection according to the inclusion criteria, two researchers (GGY and HAR) selected the articles and reviewed all possible studies independently. If the eligibility of a study was controversial between them, it was resolved by consulting the third researcher (HX).

### 2.3. Data Extraction and Quality Assessment

The following data from each study was extracted independently by two researchers (GGY and HAR) from the full text: the first author's name, publication year, study type, study location, participants characteristics (number, mean/median age at baseline, sex composition), duration of follow-up, the proportion of diabetes mellitus (DM) and hypertension, levels of serum/plasma sST2, main end point, and multivariable-adjusted HR (95% CI).

To evaluate the risk of bias and the quality of eligible studies, the Newcastle-Ottawa Scale (NOS) was used by two researchers (GGY and HAR) independently [12]. This scale included items related to selection of participants, comparability of study design and analysis, inclusion and exclusion criteria at baseline, follow-up years, confounding adjustment on the multivariate analysis, and outcome ascertainment. The accumulated scores categorized studies into 6-9 points or less than 6 points.

### 2.4. Data Analysis and Statistical Methods

The pooled adjusted HRs with 95% CIs were calculated to evaluate the strength of the association between sST2 levels and adverse outcomes in CKD patients. The Higgins' *I*^2^ statistic was further used to evaluate the heterogeneity of the studies. The included studies were considered as having low likelihood of heterogeneity when *I*^2^ < 50%. The fix-effects model was used if there is no significant heterogeneity among included studies; otherwise, the random-effects model was used. The publication bias was evaluated by using Begg's and Egger's test. Subgroup analysis and meta-regression on potential sources of heterogeneity of studies such as stage of kidney disease were also performed. The statistical analyses were done using STATA 12 (Stata Corp, College Station, TX, USA) (*p* < 0.05).

## 3. Results

### 3.1. Literature Search and Characteristics of Selected Studies

**Figure 1:**
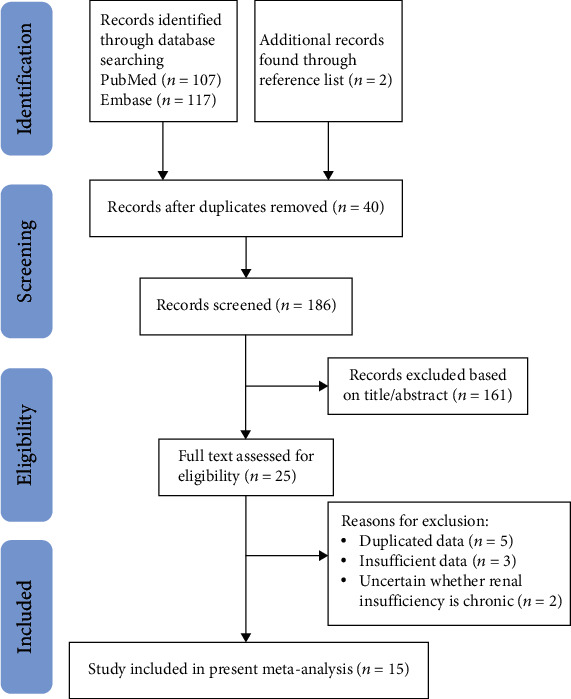
Flow diagram of the search strategy.

A total of 107 studies from PubMed, 117 studies from Embase, and two studies from the reference lists were identified initially up to August 2020. After exclusion of duplicate and nonrelevant studies, 25 studies were reviewed in full text. Another ten studies were excluded for other reasons (Figure 1), and fifteen studies met our inclusion criteria and were finally included in the meta-analysis [8, 9, 13–25].

Among the 15 studies, eight studies specifically reported dialysis patients [9, 13, 14, 17–19, 22, 24], five studies specifically reported predialysis [8, 15, 20, 21, 23], and two studies specifically reported kidney transplant patients [16, 25]. The main characteristics are summarized in Table 1. The concentration of sST2 was determined in plasma/serum by Enzyme-Linked Immunosorbent Assay (ELISA) method in almost all studies except for one using proteomics. Study individuals ranged from 74 to 3,314 with a total of 11,063, and the mean or median follow-up years ranged from 1.7 to 16.2. Six studies were conducted in European countries [8, 13, 15, 18, 22, 25], five from Asian countries [9, 14, 17, 19, 24], and the other four were carried out in America [16, 20, 21, 23]. In addition, the quality of five studies was high, and another ten studies was moderate (Table 2).

### 3.2. sST2 and All-Cause Mortality in CKD Patients

**Figure 2:**
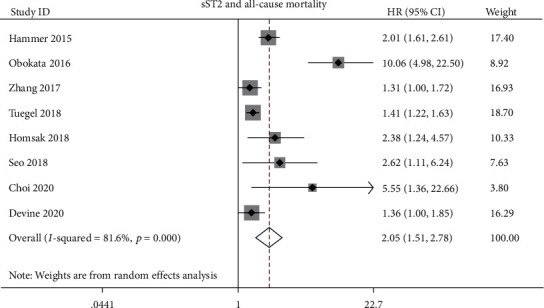
Association between sST2 and all-cause mortality in CKD patients.

Nine studies evaluated the prognostic value of sST2 for all-cause mortality [9, 13, 14, 17–20, 24, 25]. Among the nine studies, sensitivity analysis showed results from Wang et al. [9] that contributed great heterogeneity to both all-cause mortality and CVD mortality. Meta-analysis of the remaining eight studies showed increased sST2 level that was positively associated with increased all-cause mortality (HR: 2.05; 95% CI: 1.51-2.78; *p* < 0.001; Figure 2) with heterogeneity (*I*^2^: 81.6%, *p*_het_ ≤ 0.001) in CKD patients.

We also conducted subgroup analysis based on treatment, continent, DM percentage, and sample size. The subgroup analysis based on treatment showed that high sST2 levels could predict all-cause mortality in each subgroup (HR for dialysis: 2.72; 95% CI: 1.65-4.49; HR for predialysis: 1.41; 95% CI: 1.22-1.63; HR for transplant patients: 1.36; 95% CI: 1.00-1.85; Table 3). The subgroup analysis based on continent, DM percentage, and sample size showed that these factors did not influence the prognostic role of sST2 levels to all-cause mortality (Table 3).

### 3.3. sST2 and CVD Mortality in CKD Patients

**Figure 3:**
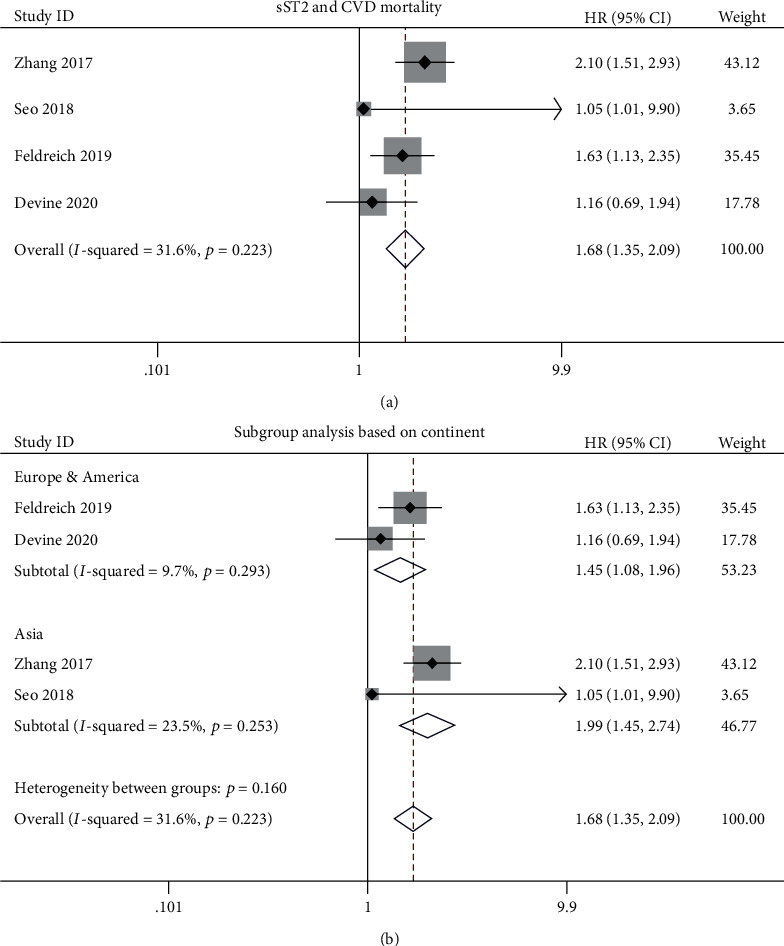
Association between sST2 and cardiovascular disease (CVD) mortality in CKD patients: (a) overall analysis; (b) subgroup analysis based on continent (Europe and America vs. Asia).

Five studies evaluated the prognostic value of sST2 for CVD mortality [9, 17, 19, 22, 25]. Meta-analysis of these studies showed high sST2 levels increased the risk of CVD mortality in CKD patients with a pooled HR of 1.68 (95% CI: 1.35-2.09; *p* < 0.001; Figure 3(a)) without significant heterogeneity (*I*^2^: 31.6%, *p*_het_ = 0.223). In subgroup analysis based on continent, high sST2 levels increased the risk of CVD mortality by 45% (HR: 1.45; 95% CI: 1.08-1.96; *p* = 0.014; *I*^2^: 9.7%, *p*_het_ = 0.293; Figure 3(b)) for CKD patients in Europe and America. More significantly, high sST2 levels increased risk of CVD mortality for CKD patients in Asia by 99% (HR: 1.99; 95% CI: 1.45-2.74; *p* < 0.001; *I*^2^: 23.5%, *p*_het_ = 0.253; Figure 3(b)).

### 3.4. sST2 and CVD Events in CKD Patients

**Figure 4:**
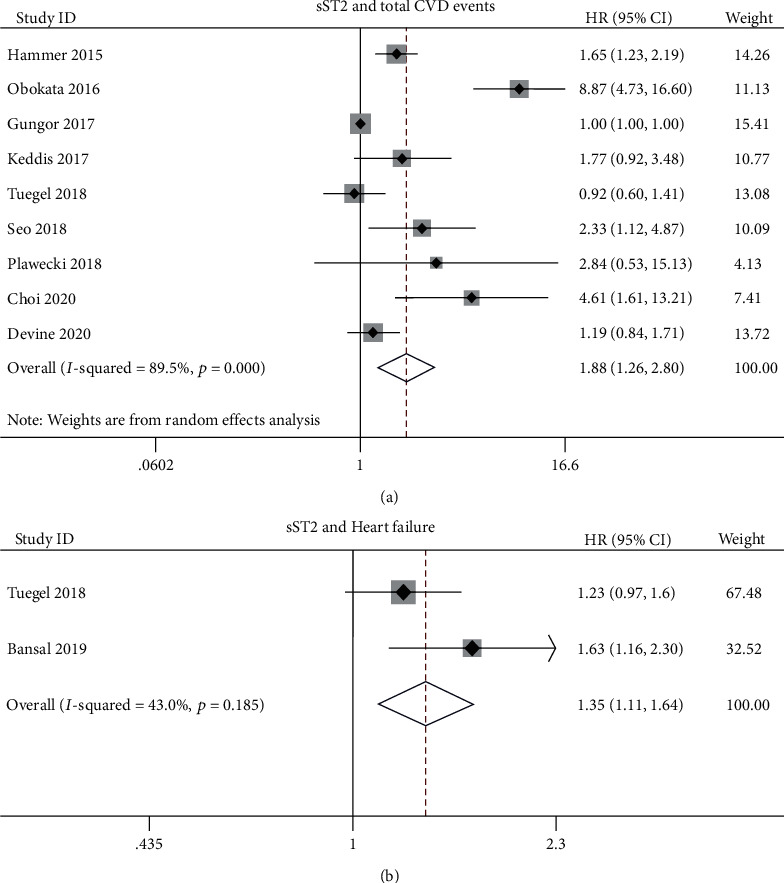
(a) Association between sST2 and total cardiovascular disease (CVD) events in CKD patients. (b) Association between sST2 and heart failure (HF) in CKD patients.

Nine studies evaluated the prognostic value of sST2 for total CVD events [8, 13–16, 19, 20, 24, 25]. When pooling data of total CVD events, we found that high sST2 levels increased the risk of total CVD events (HR: 1.88; 95% CI: 1.26-2.80; *p* = 0.002; Figure 4(a)) with heterogeneity (*I*^2^: 89.5%, *p* ≤ 0.001) in CKD patients.

Subsequently, we performed subgroup analysis based on treatment, continent, DM percentage, and sample size. The subgroup analysis based on treatment showed that high sST2 levels could predict total CVD events among dialysis patients (HR: 3.43; 95% CI: 1.43-8.24; Table 4) rather than predialysis or transplant patients. The subgroup analysis based on continent showed that high sST2 levels could predict total CVD events for CKD patients in Asia (HR: 4.64; 95% CI: 1.94-11.1; Table 4) rather than in Europe and America. The subgroup analysis based on DM percentage showed that high sST2 levels could predict total CVD events among the studies with DM proportion over median percentage (HR: 2.30; 95% CI: 1.02-5.21; Table 4).

Additionally, two studies evaluated the prognostic value of sST2 for HF [20, 21]. The elevated levels of sST2 were associated with an increased risk of HF (HR: 1.35; 95% CI: 1.1-1.64; *p* = 0.003; *I*^2^: 43.0%, *p*_het_ = 0.185; Figure 4(b)). There was only one study investigated the association between sST2 levels and the risk of atrial fibrillation (AF) among CKD patients. It showed that high levels of sST2 increased the risk of AF by 68% (HR: 1.68; 95% CI: 1.09-2.58).

### 3.5. Publication Bias

To evaluate publication bias, Begg's and Egger's tests were performed. The result of Begg's and Egger's analysis showed there was no significant publication bias for all-cause mortality (*p*_begg's_ = 0.174, *p*_egger's_ = 0.049; Supplementary Figure 1A) and CVD mortality (*p*_begg's_ = 0.308, *p*_egger's_ = 0.275; Supplementary Figure 1B).

## 4. Discussion

There were 697.5 million cases of all-stage CKD, and 1.2 million people died from CKD in 2017 Global Burden of Disease study [1]. Individuals with CKD are five to ten times more likely to die prematurely than they are to progress to ESRD [26]. The increased risk of death is mainly attributed to death from CVD [1, 2, 26]. Compared with people with normal renal function, CVD mortality is approximately 57% higher in CKD patients [27]. In MHD patients, the overall prevalence of CVD is about 71%, and approximately half of deaths are attributed to CVD [28]. However, there are currently no satisfactory biomarkers on stratifying the prognosis for life-threatening events in CKD patients. Based on such situation, it is essential to explore biomarkers that can identify high-risk patients and to eventually improve the prognosis of CKD patients. Therefore, we conducted this meta-analysis to assess sST2 in predicting all-cause mortality, CVD mortality, and CVD events among CKD patients.

\sST2 has been identified as a biomarker of cardiomyocyte hypertrophy, cardiac fibrosis, and inflammation, predicting risk of hospitalization, all-cause mortality, CVD events, and sudden death among HF and myocardial infarction patients [4, 6]. According to the 2013 American College of Cardiology Foundation/American Heart Association Guideline, sST2 was recommended as an added risk stratification factor in patients with HF [29]. Our study systematically and comprehensively evaluated the prognostic value of sST2 in CKD patients. The results of this meta-analysis revealed that elevated sST2 concentration was significantly associated with increased risk of all-cause mortality. Our results are in consistent with the previous meta-analysis which reported significant link between elevated levels of sST2 and all-cause death in MHD patients after analyzing four studies [30]. Compared with this meta-analysis, our study evaluated the predictive value of sST2 levels in a larger CKD population including dialysis patients, predialysis patients, and kidney transplant patients. In addition, we also analyzed the association between sST2 levels and CVD mortality and CVD events, revealing elevated sST2 levels could predict CVD mortality and CVD events. Several studies reported that the level of sST2 increased significantly in CKD patients and remained stable regardless of CKD stage [4, 15]. Besides this, sST2 has low biological variability and is not disturbed by age, sex, diabetes, and dialysis [8, 16, 18, 31]. These findings further indicated that sST2 is a promising prognostic biomarker for CKD patients. Interestingly, our subgroup analysis showed a significant association between increased sST2 levels and risk of all-cause mortality, CVD mortality, and total CVD events among CKD patients from Asia, whereas patients from Europe and America showed less significant results. This difference might come from specific demographic characteristics and heterogeneity of the study population. All five Asian studies were conducted among CKD patients on dialysis, whereas all studies focused on predialysis CKD patients were conducted in Europe and America. Therefore, further studies on the prognosis of sST2 among predialysis CKD patients in Asia are required.

In acute HF patients combined with renal failure, the level of sST2 was correlate positively with NT-proBNP and showed better short-term prediction outcomes than NT-proBNP [32]. Among ESRD patients with elevated level of NT-proBNP, sST2 could accurately recognize individuals at high risk of CVD mortality and HF [18]. When combined multiple risk markers, sST2 improved risk stratification effectively and could better assess the adverse outcome risk both in MHD and predialysis CKD patients [8, 14, 17, 18]. Some studies have showed the relationship between elevated sST2 levels and abnormal cardiovascular function, including left ventricular diastolic dysfunction, increased cardiac remodeling, abnormal cardiac mechanics, and endothelial dysfunction which were recognized through analyzing echocardiography and brachial artery ultrasound data in ESRD patients [15, 19]. In the future, the combination of sST2 with other markers such as NT-proBNP, cardiovascular ultrasound data may provide better prognostic value in CKD patients.

The strengths of this meta-analysis are that a number of CKD patients were included and subgroup analysis concerning multiple factors was conducted. However, there are some limitations that should be noted. First, the included studies were cohort studies. Because of its nature, this type of studies may have more confounders such as baseline patient characteristics and incomplete data collection, which might affect the stability of results. Second, although almost all HR values included are adjusted from multivariate analyses, there were still residual confounding factors cannot be excluded, and the different analyses may cause different results. Third, the pooled analysis of included studies showed high heterogeneity, and the number of included studies was small so that we could not conduct more subgroup analyses. Also, the sST2 levels varied across studies with different definition of elevation, and we could not report the boundary value in pooled studies. The retrospective nature of included studies also confers publication bias although the included studies are of high quality from NOS quality scare. Finally, the definition of CVD events in each study was inconsistent and might cause confounding bias on the pooled HR values of the risk of CVD events.

## 5. Conclusion

Our results show that high levels of sST2 could predict the all-cause mortality, CVD mortality, and CVD events in CKD patients. Larger cohort studies and researches are needed to identify high-risk individuals and explore more precise treatment in the future.

## Figures and Tables

**Table 1:** Baseline characteristics of included studies.

Author year	Country	Study population	Subjects (*n*)	Male (%)	DM (%)	HTN (%)	CVD (%)	Age (year)	Follow-up (year)	ST2 (ng/mL)	End-point event
Hammer 2015	Germany	HD	1196	54.0%	100.0%	NA	NA	66.0 ± 8.3	4.0	25.0 (median)	All-cause mortality Total CVD events
Obokata 2016	Japan	HD	423	68.8%	46.6%	84.6%	16.5%	66.0 ± 12.0	2.1 ± 0.4	0.3 (median)	All-cause mortality Total CVD events
Zhang 2017	China	HD	414	61.6%	22.9%	94.0%	9.2%	61.8 (median)	1.9 (median)	26.9 (median)	All-cause mortality CVD mortality
Gungor 2017	Turkey	Predialysis CKD	228	NA	26.3%	23.7%	18.9%	NA	2.0	NA	Total CVD events
Keddis 2017	US	Txp	200	59.7%	38.5%	94.5%	33.5%	53.0 (median)	2.3 (median)	27.8 (median)	Total CVD events
Tuegel 2018	US	Predialysis CKD	883	56.0%	43.0%	87.0%	40.0%	57.0 ± 15.0	3.1 (median)	NA	All-cause mortality HF events Total CVD events
Homsak 2018	Slovenia	HD	123	58.5%	36.6%	91.0%	26.0%	66.0 (median)	2.3 (median)	28.0 (median)	All-cause mortality
Seo 2018	Korea	HD	182	57.7%	56.0%	80.8%	14.3%	NA	1.7 (median)	80.7 ± 59.2	All-cause mortality CVD mortality Total CVD events
Plawecki 2018	France	Predialysis CKD	218	64.0%	NA	NA	NA	68.3 (median)	3.0 (median)	29.5 (median)	Total CVD events
Bansal 2019	US	Predialysis CKD	3314	54.0%	47.0%	NA	26.0%	57.5 (median)	7.9 (median)	NA	HF events
Lamprea-Montealegre 2019	US	Predialysis CKD	3053	54.8%	48.1%	NA	28.0%	57.1 ± 11.2	8.0 (median)	NA	AF events
Feldreich 2019	UK	HD	183	45.0%	25.0%	NA	19.0%	63.0 ± 14.0	2.6 (median)	NA	CVD mortality
Choi 2020	Korea	PD	74	63.5%	28.4%	NA	13.5%	53.9 ± 11.8	3.2 (median)	75.0 ± 26.6	CVD mortality Total CVD events
Devine 2020	UK	Txp	367	63.8%	13.6%	80.4%	21.8%	47.0 (median)	16.2 (median)	33.1 (median)	All-cause mortality CVD mortality Total CVD events
Wang 2020	China	HD	205	61.0%	NA	NA	NA	59 (median)	3	16.0 (median)	All-cause mortality CVD mortality

NA: not available; HD: hemodialysis; CKD: chronic kidney disease; HDF: hemodiafiltration; Txp: transplant patients; PD: peritoneal dialysis; CVD: cardiovascular disease; HF: heart failure; AF: atrial fibrillation; DM: diabetes mellitus.

**Table 2:** The quality assessment of included study using the Newcastle–Ottawa scale.

Study	Selection				Comparability		Outcome			TOTAL	Quality
	REC	SNEC	AE	DO	SC	AF	AO	FU	AFU		
Hammer 2015	1	1	1	1	0	0	1	1	1	7	High
Obokata 2016	1	1	1	1	0	0	1	0	1	6	Moderate
Zhang 2017	1	1	1	1	0	0	1	0	1	6	Moderate
Gungor 2017	1	1	1	1	0	0	1	0	1	6	Moderate
Keddis 2017	1	1	1	1	0	0	1	0	1	6	Moderate
Tuegel 2018	1	1	1	1	0	0	1	0	1	6	Moderate
Homsak 2018	1	1	1	1	0	0	1	0	1	6	Moderate
Seo 2018	1	1	1	1	0	0	1	0	1	6	Moderate
Plawecki 2018	1	1	1	1	0	0	1	0	1	6	Moderate
Bansal 2019	1	1	1	1	0	0	1	1	1	7	High
Lamprea-Montealegre 2019	1	1	1	1	0	0	1	1	1	7	High
Feldreich 2019	1	1	1	1	0	0	1	1	1	7	High
Choi 2020	1	1	1	1	0	0	1	0	1	6	Moderate
Devine 2020	1	1	1	1	0	0	1	0	1	6	Moderate
Wang 2020	1	1	1	1	0	0	1	1	1	7	High

REC: representativeness of the exposed cohort; SNEC: selection of the nonexposed cohort; AE: ascertainment of exposure; DO: demonstration that outcome of interest was not present at start of study; SC: study controls for age and sex; AF: study controls for any additional factors (chemoradiotherapy, curative resection); AO: assessment of outcome; FU: follow-up long enough (36 M) for outcomes to occur; AFU: adequacy of follow-up of cohorts. “1” means that the study is satisfied the item, and “0” means the opposite situation.

**Table 3:** Subgroup analysis for the prognostic value of sST2 on all-cause mortality.

Subgroups	All-cause mortality			
	*N*	HR (95% CI)	*p* value	Heterogeneity *I*^2^ (%), *p*
Overall	8	2.05 (1.51-2.78)	<0.001	81.6%, ≤0.001
Treatment				
Dialysis	6	2.72 (1.65-4.49)	≤0.001	83.0%, ≤0.001
Predialysis	1	1.41 (1.22-1.63)	≤0.001	—
Txp	1	1.36 (1.00-1.85)	0.050	—
Continent				
Europe and America	4	1.63 (1.29-2.06)	≤0.001	66.4%, 0.038
Asia	4	3.53 (1.17-10.66)	0.025	89.5%, ≤0.001
DM proportion				
<Median percentage	4	1.59 (1.14-2.24)	0.007	53.2%, 0.093
≥Median percentage	4	2.60 (1.51-4.47)	0.001	90.1%, ≤0.001
Sample size				
<400	4	2.11 (1.26-3.53)	0.005	55.1%, 0.083
≥400	4	2.08 (1.35-3.20)	0.001	90.4%, ≤0.001

*N*, number of studies; HR: hazard ratio; CI: confidence interval; Txp: transplant patients; DM: diabetes mellitus.

**Table 4:** Subgroup analysis for the predictive value of sST2 on total CVD events.

Subgroups	Total CVD events			
	*N*	HR (95% CI)	*p* value	Heterogeneity *I*^2^ (%), *p*
Overall	9	1.88 (1.26-2.80)	0.002	89.5%, ≤0.001
Treatment				
Dialysis	4	3.43 (1.43-8.24)	0.006	87.8%, ≤0.001
Predialysis	3	1.00 (1.00-1.00)	0.009	0%, 0.441
Txp	2	1.31 (0.94-1.82)	0.112	6.1%, 0.302
Continent				
Europe and America	6	1.23 (0.96-1.59)	0.107	70.3%, 0.005
Asia	3	4.64 (1.94-11.10)	0.001	72.8%, 0.025
DM proportion				
<Median percentage	4	1.38 (0.93-2.06)	0.112	74.5%, 0.008
≥Median percentage	4	2.30 (1.02-5.21)	0.046	91.5%, ≤0.001
Sample size				
<400	6	1.57 (1.06-2.33)	0.023	72.7%, 0.003
≥400	3	2.30 (0.82-6.44)	0.112	94.2%, ≤0.001

CVD: cardiovascular disease; *N*: number of studies; HR: hazard ratio; CI: confidence interval; Txp: transplant patients; DM: diabetes mellitus.

## Data Availability

The data used to support the findings of this study are available from the corresponding author upon request.

## Supplementary Materials

Supplementary Materials Supplementary Figure 1: (a) Funnel plot for meta-analysis related to all-cause mortality. (b) Funnel plot for meta-analysis related to cardiovascular disease (CVD) mortality.
