# Supplementary figures and images for: Prognostic Value of Soluble Suppression of Tumorigenicity 2 in Chronic Kidney Disease Patients: A Meta-Analysis
Source: Dis Markers. 2021 Jan 25;2021:8881393. doi: 10.1155/2021/8881393 (PMC7857877; doi:10.1155/2021/8881393)

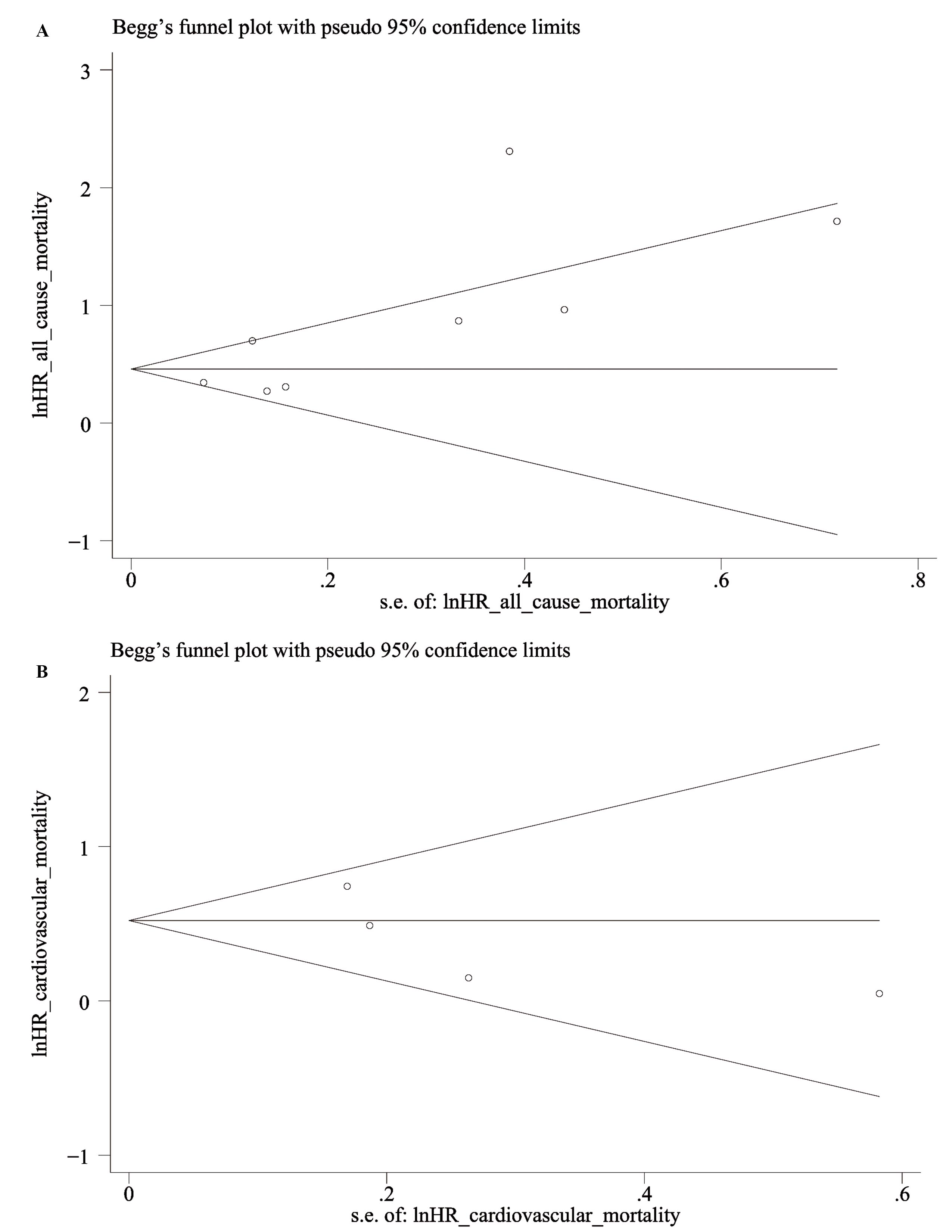

Supplement: Supplementary Materials — Supplementary Figure 1: (a) Funnel plot for meta-analysis related to all-cause mortality. (b) Funnel plot for meta-analysis related to cardiovascular disease (CVD) mortality. [file 8881393.f1.jpg]
